# Role of *dipA* and *pilD* in *Francisella tularensis* Susceptibility to Resazurin

**DOI:** 10.3390/antibiotics10080992

**Published:** 2021-08-17

**Authors:** Kendall Souder, Emma J. Beatty, Siena C. McGovern, Michael Whaby, Emily Young, Jacob Pancake, Daron Weekley, Justin Rice, Donald A. Primerano, James Denvir, Joseph Horzempa, Deanna M. Schmitt

**Affiliations:** 1 Department of Biomedical Sciences, West Liberty University, West Liberty, WV 26074, USA; kssouder@westliberty.edu (K.S.); ejbeatty@westliberty.edu (E.J.B.); scmcgovern@westliberty.edu (S.C.M.); whaby@musc.edu (M.W.); emily.young@westliberty.edu (E.Y.); jppancake2121@gmail.com (J.P.); dmweekley@westliberty.edu (D.W.); jcrice1@westliberty.edu (J.R.); joseph.horzempa@westliberty.edu (J.H.); 2 Department of Biomedical Sciences, Joan C. Edwards School of Medicine, Marshall University, Huntington, WV 25755, USA; primeran@marshall.edu (D.A.P.); denvir@marshall.edu (J.D.)

**Keywords:** *Francisella tularensis*, resazurin, DipA, PilD, tularemia, antimicrobial, antibiotic, resistance

## Abstract

The phenoxazine dye resazurin exhibits bactericidal activity against the Gram-negative pathogens *Francisella tularensis* and *Neisseria gonorrhoeae*. One resazurin derivative, resorufin pentyl ether, significantly reduces vaginal colonization by *Neisseria gonorrhoeae* in a mouse model of infection. The narrow spectrum of bacteria susceptible to resazurin and its derivatives suggests these compounds have a novel mode of action. To identify potential targets of resazurin and mechanisms of resistance, we isolated mutants of *F. tularensis* subsp. *holarctica* live vaccine strain (LVS) exhibiting reduced susceptibility to resazurin and performed whole genome sequencing. The genes *pilD* (FTL_0959) and *dipA* (FTL_1306) were mutated in half of the 46 resazurin-resistant (RZR) strains sequenced. Complementation of select RZR LVS isolates with wild-type *dipA* or *pilD* partially restored sensitivity to resazurin. To further characterize the role of *dipA* and *pilD* in resazurin susceptibility, a *dipA* deletion mutant, Δ*dipA*, and *pilD* disruption mutant, FTL_0959d, were generated. Both mutants were less sensitive to killing by resazurin compared to wild-type LVS with phenotypes similar to the spontaneous resazurin-resistant mutants. This study identified a novel role for two genes *dipA* and *pilD* in *F. tularensis* susceptibility to resazurin.

## 1. Introduction

The Centers for Disease Control and Prevention (CDC) estimates that there are nearly three million new cases of antibiotic resistant bacterial infections annually, resulting in 35,000 deaths and billions of dollars in health care costs in the United States [1]. The rise in antibiotic resistance has been attributed to over-prescription and improper use of commonplace antibiotics [2]. Despite the clinical threat posed by increasing antibiotic resistance, the development of new antibiotics has significantly slowed due to decreased profitability [3]. While there are over 40 antimicrobial agents in clinical trials, most belong to existing classes of antibiotics such as beta lactams and beta-lactamase inhibitors, tetracyclines, and aminoglycosides [4]. Each of these classes of antibiotics targets similar bacterial processes including cell wall synthesis and protein synthesis. The development of bacterial resistance against these new drugs in the future is likely since there is already selective pressure from current antibiotics. Therefore, new antibiotic targets should be explored to minimize the emergence of resistance and provide effective alternatives.

Phenoxazine-based compounds have been shown to have antimicrobial activity [5,6]. Actinomycin D, the most well-known phenoxazine-containing antibiotic, suppresses the growth of various Gram-positive and Gram-negative bacteria as well as *Mycobacterium tuberculosis* [7]. Other actinomycins, neo-actinomycins A and B, exhibit moderate antibacterial activity against methicillin-resistant *Staphylococcus aureus* (MRSA) and vancomycin-resistant *Enterococcus* (VRE) strains [8]. We previously showed the phenoxazine dye resazurin inhibits growth of the Gram-negative human pathogens *Neisseria gonorrhoeae* and *Francisella tularensis* [9,10]. Resorufin pentyl ether, an analog of resazurin, significantly reduces vaginal colonization by *N. gonorrhoeae* in a mouse model of infection [10]. However, the mechanism by which resazurin kills *F. tularensis* and *N. gonorrhoeae* has not been defined. Based on its structural similarity to other phenoxazine compounds, resazurin may function as a DNA intercalating agent [5]. Unlike other phenoxazine compounds, resazurin is non-toxic to eukaryotic cells, and its bactericidal activity is limited to only two Gram-negative bacterial species suggesting a novel mechanism of action [5,6,9]. 

In this study, we sought to identify *F. tularensis* genetic determinants involved in susceptibility to resazurin in hopes of elucidating the mode of action of this compound.

## 2. Results

### 2.1. Isolation of Resazurin-Resistant F. tularensis LVS Mutants

A common strategy bacteria employ to develop antimicrobial resistance is by mutating gene(s) associated with the mechanism of action of the antibiotic. Therefore, to identify potential targets of resazurin, we selected for mutants of *F. tularensis* LVS that were capable of growing in the presence of 22 µg/mL of resazurin. This concentration of resazurin is twenty-fold higher than the previously determined MIC of resazurin for *F. tularensis* LVS [9]. The genomes of forty-six spontaneous resazurin-resistant (RZR) mutants were sequenced and compared to wild-type LVS to identify genetic variants. Nonsynonymous mutations were identified in ten different protein-coding *F. tularensis* LVS genes with approximately 50% of the isolates possessing mutations in FTL_1306 (*dipA*) and FTL_0959 (*pilD*) (Table 1). The same *pilD* variant was observed in twenty-two of the RZR LVS isolates involving a single base pair substitution leading to a premature stop codon at position 187 of the protein sequence (Table 2). Fifteen different coding mutations were characterized in *dipA* that included single base pair substitutions, insertions, and deletions with many resulting in nonsense mutations (Table 2). Of note, one RZR LVS isolate, RZR46, contained a deletion of a single thymine residue at Position 2 in *dipA* resulting in loss of the start codon (Table 2). Western blot analysis confirmed this mutation abolished expression of DipA protein in RZR46 (data not shown). These data suggest the genes *dipA* and *pilD* play a role in *F. tularensis* LVS susceptibility to resazurin.

### 2.2. Characterizing the Role of pilD and dipA in F. tularensis Susceptibility to Resazurin

Almost all of the RZR LVS strains isolated possessed nonsynonymous mutations in multiple genes with some exceptions. The sole nonsynonymous mutation in RZR47 is in *pilD* while RZR46 contains a single nucleotide deletion resulting in loss in expression of the DipA protein (Table 2; data not shown). These findings suggest that loss of either *pilD* or *dipA* function may confer resazurin resistance with limited contributions/effects from other genes. To confirm the mutations in *dipA* and/or *pilD* were contributing to the reduced susceptibility of RZR strains to resazurin, we individually cloned wild-type copies of *dipA* and *pilD* into the *Francisella* vector pABST which contains the robust *groE* promotor of *F. tularensis* [11] and then introduced these new plasmids, pDipA and pPilD, into RZR46 and RZR47, respectively. Then, we tested the sensitivity of these strains to resazurin. We hypothesized that restoring expression of DipA and/or PilD in these RZR strains would alter their resistance phenotype. RZR46 and RZR47 containing pABST alone served as controls. The resazurin MICs for RZR46/pABST and RZR47/pABST were two-fold higher than the MIC for wild-type LVS (Table 3). Introduction of the pABST vector into the RZR strains did not alter their susceptibility to resazurin (data not shown). Complementation of RZR46 with *dipA* restored resazurin sensitivity back to wild-type with both RZR46/pDipA and LVS having MICs of 5.5 µg/mL (Table 3). In contrast, RZR47 containing the *pilD* expression construct has the same resazurin MIC as the vector control (Table 3). These data confirm a role for *dipA*, but not *pilD*, in *F. tularensis* LVS susceptibility to resazurin.

**Figure 1:**
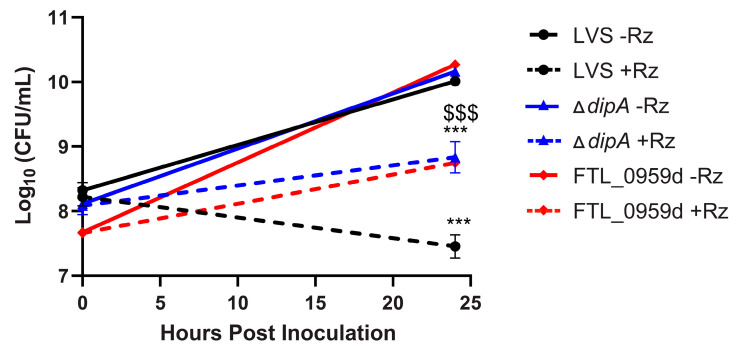
Reduced killing of FTL_0959d and Δ*dipA* by resazurin compared to wild-type LVS. Bacteria were cultivated in tryptic soy broth supplemented with 0.1% cysteine HCl (TSBc) in the presence or absence of resazurin (Rz, 1.375 µg/mL) for 24 h. Cultures were then diluted and plated to determine the number of viable *F. tularensis* LVS bacteria 24 h post inoculation. Data shown are mean ± SEM from three individual experiments. The limit of detection was 100 CFU per ml. Statistically significant differences in growth post-inoculation were determined by two-way ANOVA followed by Tukey’s multiple comparisons test (***, *p* < 0.001 comparing +Rz to −Rz for each strain; $$$, *p* < 0.001 comparing FTL_0959d and Δ*dipA* to LVS 24 h. post Rz treatment).

To individually investigate the role of *dipA* and *pilD* in resazurin susceptibility in a wild-type background, we generated a *pilD* disruption mutant (FTL_0959d) and a *dipA* deletion mutant (Δ*dipA*) in *F. tularensis* LVS. The MICs of both FTL_0959d and Δ*dipA* were two-fold higher than that of wild-type LVS and comparable to the MICs determined for the spontaneous resazurin-resistant mutants RZR46 and RZR47 (Table 3 and Table 4). We next sought to evaluate the bactericidal activity of resazurin against FTL_0959d and Δ*dipA* over time. Treatment with resazurin resulted in a significant reduction in viable bacteria after 24 h compared to untreated controls for all strains tested (Figure 1). However, significantly more FTL_0959d and Δ*dipA* bacteria were recovered 24 h post resazurin treatment compared to wild-type LVS (Figure 1). These data suggest that neither DipA nor PilD are targets of resazurin, but might be involved in the uptake and metabolism of this compound by *F. tularensis* LVS.

## 3. Discussion

The clinical threat posed by antibiotic resistant bacterial infections highlights the need to identify and apply novel antimicrobial therapies [2]. Resazurin exhibits antibacterial activity against *F. tularensis* and *N. gonorrhoeae*, but its mode of action has not been defined [9,10]. Here, we screened for resazurin-resistant mutants of *F. tularensis* LVS to identify genetic determinants of resazurin susceptibility. Whole genome sequencing of RZR isolates identified nonsynonymous mutations in ten different protein-coding genes. None of the genes identified in this screen were previously described to play a role in *F. tularensis* resistance to other clinically relevant antibiotics such as aminoglycosides, tetracyclines and fluoroquinolones [12,13]. Therefore, these data suggest resazurin has a unique mechanism of action.

In particular, two genes, *dipA* and *pilD,* were mutated in the majority of the RZR LVS clones isolated and sequenced. Complementation of RZR46 with *dipA* restored sensitivity to resazurin comparable to wild-type LVS while expression of *pilD* in RZR47 did not. However, both a laboratory generated *pilD* disruption mutant and *dipA* deletion mutant had higher MICs compared to wild-type LVS similar to the spontaneous RZR mutants. The failure to restore resazurin sensitivity in RZR47 upon complementation with wild-type *pilD* may be due to a dominant negative effect of the original *pilD* mutation or another intergenic/undetected variant in RZR47 may be contributing to the resazurin resistance. Regardless, less killing of FTL_0959d and Δ*dipA* was observed over 24 h following resazurin treatment compared to wild-type LVS confirming a role for both *dipA* and *pilD* in resazurin susceptibility.

DipA is a 353 amino acid protein predicted to form a surface complex with other outer membrane proteins to facilitate translocation of virulence factors that interact with host cells [14]. Most of the *dipA* mutations characterized in this study occurred within the Sel1-like repeat domains (amino acids 95–170 and 192–263) which are known to be required for the biological function of DipA [14]. These structural domains provide a scaffold for protein–protein interactions [15]. DipA has been shown to associate with FopA, a *F. tularensis* outer membrane protein with noted homology to the OmpA porin [14]. OmpA has been shown to play a role in antibiotic resistance in other Gram-negative pathogens including *Escherichia coli* and *Acinetobacter baumannii* [16,17]. Given the correlation between DipA mutations and resazurin resistance in this study, it is possible that DipA plays a role in the stability or function of FopA allowing for uptake of resazurin. Without functional DipA, less resazurin may be taken up by *F. tularensis* LVS resulting in increased resistance to resazurin. Interestingly, none of the resazurin resistant strains isolated and sequenced in this study contained mutations in the LVS gene encoding FopA. The role of DipA and FopA in uptake of resazurin by *F. tularensis* is currently being investigated.

The PilD protein was also shown to contribute to *F. tularensis* susceptibility to resazurin. PilD is an inner membrane peptidase responsible for processing major and minor (pseudo) pilins involved in the formation of Type IV pili and assembly of a Type II protein secretion system [18,19]. In *P. aeruginosa*, PilD has also been shown to play a role in the export of select enzymes including alkaline phosphatase, phospholipase C, elastase, and exotoxin A [20]. Although the exact function of PilD in *F. tularensis* has not been fully investigated and defined, it is possible PilD may play a role in the processing and/or export of a protein that is a target of resazurin. Mutation of *pilD* would thus affect processing or export of the antibiotic target rendering *F. tularensis* less susceptible to resazurin. The exact role PilD plays in *F. tularensis* susceptibility to resazurin is under further investigation. 

As *F. tularensis* is not the only bacterium susceptible to resazurin, we wondered whether *pilD* (FTL_0959) and *dipA* (FTL_1306) homologs were present in *N. gonorrhoeae* and played similar roles as described here. A BLAST search [21] against the National Center for Biotechnology Information database of non-redundant protein sequences identified an A24 family peptidase (WP_003689814.1) and a SEL1-like repeat protein (MBS5742101.1) in *Neisseria* that were only 43% and 26% identical to *F. tularensis* PilD and DipA, respectively. The low homology between these proteins suggests that PilD and DipA have unique roles in *F. tularensis* susceptibility to resazurin. It also remains unknown whether DipA and PilD function in the same pathway to mediate *F. tularensis* killing by resazurin. The proposed function of DipA in transport and PilD in pilin processing suggests that they function separately from one another, but the relationship between DipA and PilD in mediating susceptibility to resazurin is currently being explored. Overall, mutations in *pilD* and *dipA* failed to confer complete resistance to resazurin. The growth of both FTL_0959d and Δ*dipA* was still inhibited by resazurin and the MICs for these mutants were only two-fold higher than wild-type LVS. An alternative method to identify potential antibiotic targets from the one used in this study is high-throughput transposon sequencing (Tn-Seq) [22,23,24]. In this approach, a library of LVS transposon mutants would be generated and screened for resazurin resistance [25]. Then, next-generation sequencing of the transposon insertion would be performed and the insertion sites would be mapped to the LVS genome. Further understanding of resazurin’s mode of action and resistance mechanisms will help guide the development of resazurin derivatives with more potent activity as well as new therapeutic strategies to combat *Francisella* infections.

## 4. Materials and Methods

### 4.1. Bacterial Strains and Growth Conditions

Bacterial strains used in this study are listed in Table 5. For cultivation of *F. tularensis* LVS strains, frozen stock cultures were plated on chocolate II agar and incubated at 37 °C with 5% CO_2_ for 2–4 days. These bacteria were then used to inoculate TSBc (trypticase soy broth (BD Biosciences) containing 0.1% l-cysteine hydrochloride monohydrate (Fisher)). *Escherichia coli* 5-α (New England Biolabs) bacteria cultivated on LB agar were used to inoculate LB broth. All broth cultures were incubated at 37 °C with agitation. When required, kanamycin (35 μg/mL for *E. coli*; 10 μg/mL for *F. tularensis*) and hygromycin (250 μg/mL) were supplemented into the media. 

### 4.2. Selection of Resazurin-Resistant (RZR) LVS Mutants and Whole Genome Sequencing

Mutants resistant to resazurin were obtained by plating suspensions containing approximately 5 × 10^8^ CFU of *F. tularensis* LVS bacteria on chocolate II agar supplemented with 11 µg/mL resazurin sodium salt (Acros Organics, dissolved in water) [9]. This concentration of resazurin is ten-fold higher than the previously determined minimal inhibitory concentration (MIC) of resazurin [9]. Colonies that formed on plates containing 11 µg/mL resazurin were then replica-plated onto 22 µg/mL resazurin. Forty-six resazurin-resistant (RZR) LVS clones that grew in the presence of 22 µg/mL resazurin were selected for further analysis. Genomic DNA was isolated using the Bacterial Genomic DNA Isolation Kit (Norgen Biotek Corp., Thorold, ON, Canada) per the manufacturer’s instructions. Sequencing libraries were prepared using Nextera XT DNA Library Preparation Kits (Illumina, San Diego, CA, USA) and individual libraries were indexed with barcodes using Nextera XT v2 Index Kits (Illumina, San Diego, CA, USA) in the Marshall University (MU) Genomics Core Facility. Library quality and size distribution was assessed on an Agilent Bioanalyzer equipped with High Sensitivity DNA Chips. Libraries were quantitated by Qubit fluorimetry. High throughput sequencing (2 × 100 bp paired-end) was performed on an Illumina HiSeq1500 in Rapid Run mode in the Genomics Core. This approach allowed for approximately 150× coverage of each individual bacterial genome.

Sequencing reads were trimmed to remove Illumina adapters and low quality base calls using Trimmomatic version 0.36 [27]. Trimmed reads were aligned to the *Francisella tularensis* reference genome (NCBI accession number AM23362.1) using BWA version 0.7.12 [28] and sorted and indexed using picard tools version 2.9.4. Variant calling was performed following the GATK version 3.8.0 pipeline [29]. Briefly, duplicates were marked with the MarkDuplicates tool and per-sample variants identified using HaplotypeCaller. Variants were then called across the entire sample set using the GenotypeGVCFs tool with the sample-ploidy option set to one. Variant calls with quality score below 20 were filtered out using the VariantFiltration tool. Variants were exported to tab-delimited format using VariantsToTable. After the conclusion of these GATK pipeline steps, variants were annotated to determine their effects on the protein sequence using snpEff version 4.3q [30].

### 4.3. Construction of dipA and pilD Complemented Strains and Mutants

Primers and plasmids used in this study are listed in Table 5. To complement RZR LVS mutants with either wild-type *dipA* or *pilD*, *F. tularensis* LVS chromosomal DNA extracted from stationary-phase broth cultures using the Bacterial Genomic DNA Isolation Kit (Norgen Biotek Corp.) served as a template for amplification of *dipA* (FTL_1306) and *pilD* (FTL_0959). Primer pairs dipA_F/dipA_R and pilD_F/pilD_R were used to PCR-amplify *dipA* and *pilD*, respectively. The dipA amplicon was cloned into the EcoRI-NheI site of pABST [24] to generate the plasmid, pDipA. The pilD amplicon was cloned into the EcoRI-NdeI site of pABST to create the plasmid, pPilD. Both pDipA and pPilD were introduced into select *F. tularensis* LVS resazurin-resistant mutant strains by electroporation and selection on chocolate II agar supplemented with 10 μg/mL kanamycin.

The *pilD* disruption mutant (FTL_0959d) was generated using an unstable, integrating suicide vector, pJH1, as previously reported [31,32]. An internal 470 base pair region of FTL_0959 (*pilD*) was amplified by PCR using the primers F_0959d and R_0959d. This amplicon was digested with BamHI and SphI and then cloned into pJH1 that had been digested with these same enzymes, which produced pJH1-0959d. This disruption construct was mobilized into *F. tularensis* LVS by triparental mating [31]. Hygromycin-resistant colonies were screened by PCR using the primers pJH1-conf and 1F_pilD which are specific for the vector-portion of pJH1-FTL_0959 and pilD respectively, to confirm disruption of the *pilD* gene.

A *F. tularensis* LVS *dipA* (FTL_1306) deletion mutant was generated using pJH1 and the I-SceI endonuclease as described previously [26]. To begin, the *dipA* deletion construct pJH1-ΔdipA was generated using splicing by overlap-extension PCR. Regions (~500 bp) upstream and downstream of the *dipA* sequence targeted for deletion were amplified by PCR using the primer pairs 1F_dipA with 2R_dipA and 3F_dipA with 4R_dipA, respectively. The resulting amplicons contained regions of overlap and served as template DNA for a second PCR reaction using primers 1F_dipA and 4R_dipA. The resulting 1176 bp fragment was digested with PstI and SphI and then ligated into pJH1, which had been digested with the same enzymes, to produce pJH1-ΔdipA. This vector was then transferred into *F. tularensis* LVS by tri-parental mating [31]. Merodiploid strains were recovered and transformed with pGUTS by electroporation to allow for expression of I-SceI which causes a double-stranded break forcing recombination and allelic replacement [26]. Colonies resistant to kanamycin were screened by PCR for deletion of *dipA* using primers 1F_dipA and 4R_dipA. To cure pGUTS, the *F. tularensis* Δ*dipA* strains were repeatedly sub-cultured in TSBc, diluted, and plated to a density of 100 to 300 colony forming units CFU per chocolate II agar plate. Plates were incubated for at least 3 days at 37 °C, 5% CO_2_ and colonies that formed were replica plated onto chocolate II agar supplemented with and without 10 μg/mL kanamycin to select against colonies harboring pGUTS. Those colonies sensitive to kanamycin were isolated and again tested for sensitivity to this antibiotic. The resulting LVS strain was named Δ*dipA*. Western blot analysis using an anti-DipA antibody [14] confirmed loss in expression of the DipA protein in LVS Δ*dipA* (data not shown). 

### 4.4. Agar Dilution Susceptibility Testing 

Minimum inhibitory concentrations (MICs) were determined by the agar dilution method using chocolate II agar according to CLSI guidelines [33]. Briefly, *F. tularensis* LVS bacteria were cultivated overnight in TSBc, diluted to an OD600 of 0.3 (approximately 5 × 10^8^ CFU/mL), and then diluted to 1 × 10^6^ CFU/mL. Ten microliters of this suspension (1 × 10^4^ CFU) were plated onto chocolate II agar containing a series of two-fold dilutions of resazurin. Following incubation (37 °C with 5% CO_2_ for 48–72 h), the MIC reported for each *F. tularensis* strain was the lowest concentration of resazurin that completely inhibited the growth of bacteria on the agar plate. No strain differed by more than one dilution in 3–5 tests.

### 4.5. Time Kill Assays

*F. tularensis* LVS overnight broth cultures were used to inoculate TSBc containing 1.375 µg/mL resazurin. Immediately following inoculation and 24 h later, cultures were serially diluted and plated onto chocolate II agar. Plates were incubated at 37 °C, 5% CO_2_ and individual colonies were enumerated. The limit of detection was 100 CFU/mL. 

### 4.6. Statistical Analyses

Data were analyzed for significant differences using GraphPad Prism software (GraphPad Software Inc., La Jolla, CA, USA). The statistical tests are indicated in the figure legend.

## Figures and Tables

**Table 1:** Protein-coding genes containing nonsynonymous mutations in 46 resazurin-resistant *F. tularensis* isolates sequenced.

*F. tularensis* Gene Name	Number of Resazurin-Resistant Isolates Containing Gene Mutation
FTL_1306 (*dipA*)	24
FTL_0959 (*pilD*)	22
FTL_1666	5
FTL_0073	4
FTL_0358	2
FTL_0610	2
FTL_0132	1
FTL_0315	1
FTL_1634	1
FTL_1654	1

**Table 2:** Description of *dipA* and *pilD* mutations found in sequenced resazurin-resistant (Rzr) *F. tularensis* LVS isolates.

Mutation Type	Nucleotide Change	Protein Change
DipA		
Frameshift and start lost	1_19delATGAAAGCTAAATTTATAA	Start lost
Frameshift and start lost	2delT	Start lost
Stop gained	169G > T	Glu57 *
Stop gained	211G > T	Glu71 *
Stop gained	268G > T	Glu89 *
Stop gained	310G > T	Glu104 *
Frameshift and stop gained	342_346delGGTAG	Lys114Xfs6 *
Frameshift and stop gained	349_350delCA	Gln117Lysfs6 *
Frameshift and stop gained	350_353delAAAA	Lys119Valfs2 *
Frameshift and stop gained	387_396delTTTGGGCTAT	Asn129Lysfs8 *
Stop gained	578 C > A	Ser193 *
Stop gained	655C > T	Gln219 *
Stop gained	676G > T	Glu226 *
Stop gained	723T > A	Tyr240 *
Frameshift and stop gained	751_752insA	Tyr251 *
PilD		
Stop gained	532G > T	Gly178X

* indicates stop codon.

**Table 3:** Resazurin MIC for *dipA* and *pilD* complemented *F. tularensis* resazurin-resistant strains.

Strain	MIC (μg/mL) ^1^
LVS	5.5
Rzr 46/pABST	11
Rzr 47/pABST	11
Rzr 46/pDipA	5.5
Rzr 47/pPilD	11

^1^ MICs are mode values from at least six independent determinations.

**Table 4:** MIC of resazurin for *dipA* and *pilD F. tularensis* LVS mutants.

Strain	MIC (μg/mL) ^1^
LVS	5.5
Δ*dipA*	11
FTL_0959d	11

^1^ MICs are mode values from at least three independent determinations.

**Table 5:** Description of strains, plasmids, and primers used in this study.

Plasmid, Primer, or Strain	Description or Sequence	Source or Reference
*F. tularensis* strains		
LVS	*F. tularensis* subsp. holarctica live vaccine strain	Karen Elkins
RZR46	Spontaneous resazurin-resistant LVS mutant containing deletion of single thymine residue at position 2 in *dipA*	This study
RZR47	Spontaneous resazurin-resistant LVS mutant containing 532G > T substitution in *pilD*	This study
Δ*dipA*	LVS *dipA* deletion mutant	This study
FTL_0959d	LVS *pilD* disruption mutant	This study
*E. coli* strains		
DH5α	*fhuA2 Δ(argF-lacZ)U169 phoA glnV44 Φ80 Δ(lacZ)M15 gyrA96 recA1 relA1 endA1 thi-1 hsdR17*	NEB ^1^
Plasmids		
pABST	pFNLTP8 with *F. tularensis* LVS groE promoter	[11]
pDipA	pABST containing *dipA* under control of *groE* promoter	This study
pPilD	pABST containing *dipA* under control of *groE* promoter	This study
pJH1	pMQ225 with the I-SceI restriction site	[26]
pGUTS	pGRP with I-SceI under the control of FGRp	[26]
pJH1ΔdipA	pJH1 with the left and right 500-bp flanking regions of *dipA* cloned into the PstI-SphI site of pJH1	This study
pJH1-0959d	pJH1 with the central 470 base pair region of FTL_0959	This study
Primers		
1F_dipA	5′-CATGCTGCAGGTAGTGTTTGGATCTTTTGTATTAGCAG -3′	IDT ^2^ This study
2R_dipA	5′-ATAATAAGTCGACGGTACCACCGGTAATTATAAATTTAGCTTTCATCTATTTCTCCTG-3′	IDT This study
3F_dipA	5′-ACCGGTGGTACCGTCGACTTATTATTACTACCAAAGAGCAGCAAAG-3′	IDT This study
4R_dipA	5′-CATGGCATGCCATCACCACATTTAGAACTATTGGC-3′	IDT This study
F_0959d	5′-CATGGGATCCGGGATTGATAAGCAACAATCTCCAC-3′	IDT This study
R_0959d	5′-CATTGCATGCCCAAAGCCTTCTTTACCTGTAAGG-3′	IDT This study
dipA_F	5′-CATGGAATTC GTGACGCTTGATGTTTTTGTATTGCAGG-3′	IDT This study
dipA_R	5′-CATGGCTAGCGCGCTATTTAGAAGTCACCGCATTTTG-3′	IDT This study
pilD_F	5′-ATCGGAATTCATGTTGTTATATATCGAGTGCCAAATAAAC-3′	IDT This study
pilD_R	5′-ATCACATATGTTATACATAGATATTATCTTTAGTCAGTAG ATAAAAAAATGTTG-3′	IDT This study
pJH1_conf	5′-CTGATTTAATCTGTATCAGGCTGAAAATC-3′	IDT This study

^1^ NEB—New England Biotechnologies. ^2^ IDT—Integrated DNA Technologies.

## Data Availability

All sequencing data have been submitted to the Sequence Read Archive at the National Center for Biotechnology Information (NCBI) and are accessible via project number PRJNA748943.
